# Association between Maternal Plasma Ferritin Level and Infants’ Size at Birth: A Prospective Cohort Study in Rural Bangladesh

**DOI:** 10.1080/16549716.2020.1870421

**Published:** 2021-01-19

**Authors:** Syed Moshfiqur Rahman, Md. Shahjahan Siraj, Mohammad Redwanul Islam, Anisur Rahman, Eva-Charlotte Ekström

**Affiliations:** a International Maternal and Child Health, Department of Women’s and Children’s Health, Uppsala University, Uppsala, Sweden; b Maternal and Child Health Division, International Centre for Diarrhoeal Disease Research, Dhaka, Bangladesh

**Keywords:** Ferritin, iron status, pregnancy, Bangladesh, size at birth

## Abstract

**Background**: Iron supplementation in pregnancy is recommended by the WHO to prevent a major public health problem, namely, maternal iron deficiency and its consequences. There are gaps in the existing evidence regarding maternal and neonatal benefits and harms of universal iron supplementation.

**Objective**: To evaluate the association between maternal iron status during pregnancy and infant size at birth (birth weight and length).

**Method**: This present prospective cohort study was nested in a food and micronutrient supplementation trial conducted in Matlab (MINIMat study), rural Bangladesh. We randomly selected 573 women recruited into the MINIMat study from January – December 2002 who delivered singletons with available birth anthropometric information. The plasma ferritin of each mother was measured at gestational week 14 (GW14; before the start of micronutrient supplementation) and at week 30 (GW30).

**Results**: Multivariable linear regression revealed no association between plasma ferritin at GW14 and birth weight. However, newborns of women in the highest tertile of plasma ferritin at GW30 (median = 29 µg/L) had on average a 93-gm lower birth weight (95% CI: −172, – 14; *p *= 0.021) than the newborns of women in the lowest tertile (median = 8 µg/L). Logistic regression showed that odds of low birth weight were approximately two times higher [odds ratio (OR) = 2.27; 95% CI: 1.40, 3.67] among those with mothers in the highest ferritin tertile than in the lowest tertile at GW30. No association was found between maternal plasma ferritin and birth length.

**Conclusion**: We observed an inverse association between high plasma ferritin in the last trimester (GW30) and birth weight but not birth length. The results suggested that elevated plasma ferritin in pregnancy could have an untoward effect on birth weight.

## Background

Iron (Fe) is essential for physiological functions, including hemoglobin (Hb) synthesis, and cell growth and development [1]. Iron deficiency results from depletion of stored iron. Increased iron demand during pregnancy can worsen this, resulting in iron-deficient erythropoiesis and, eventually, iron deficiency anemia [2]. If the body iron store is deficient at conception, it is unlikely that dietary iron would be able to match the pregnancy-induced increase in demand [3]. Therefore, assessment of body iron status during pregnancy is crucial. While ferritin is the most commonly used indicator [4], providers often rely on Hb levels to assess iron deficiency at the population level. This use of Hb levels is problematic for two reasons. First, decreased Hb levels can result from causes other than iron deficiency [5,6]. Second, physiological expansion of plasma volume during pregnancy leads to a lowering of hemoglobin (Hb) concentration, irrespective of the iron status. However, the appropriate size of the iron store needed during pregnancy to ensure optimal outcomes for both mothers and neonates remains unknown [7].

Several studies have evaluated the associations between maternal iron status and fetal iron status [8] and growth [9,10]. However, the findings remain inconsistent. A recent review suggests that low and high hemoglobin concentrations in early pregnancy may lead to a heightened risk of adverse birth outcomes such as low birth weight, preterm birth, and stillbirth. However, the risk of adverse birth outcome was weaker or non-significant when Hb was measured in the second or third trimester [11]. The study also reported less evidence of the association between maternal iron status assessed by serum ferritin in different trimesters and adverse birth outcomes [11].

Most of the previous studies evaluating the iron status and birth outcome used hemoglobin level as an indicator of iron status, and few studies have evaluated serum ferritin as an indicator of iron status [11,12]. Additionally, studies that assessed the health implications of maternal iron status are commonly from developed countries. There is a dearth of studies from low-income countries, and thus far, there is no available study on the relationship between maternal ferritin levels and pregnancy outcomes in Bangladesh. Therefore, we aimed to evaluate the association between maternal iron status assessed by plasma serum ferritin levels during early and late gestation of pregnancy and infant size at birth.

## Methods

### Study site, design and participants

This study was carried out in Matlab, a rural subdistrict of Bangladesh, where the International Centre for Diarrhoeal Disease Research in Bangladesh (icddr,b) has been operating a Health and Demographic Surveillance System (HDSS) since 1966. The study was nested into a larger study named Maternal and Infant Nutrition Intervention in Matlab (MINIMat Trial, reg#ISRCTN16581394) that recruited 4,436 pregnant women from November 2001 to October 2003. The design and procedures of the MINIMat trial have been reported elsewhere [13]. This prospective cohort study used information from a group of 1000 women who were randomly selected from 2119 recruited in the MINIMat trial from January to December 2002. All women were given computer generated, random numbers which were subsequently used for selection. In total, 573 women who delivered singletons with available birth anthropometric measurements and data on plasma ferritin levels in the early and late gestation periods of pregnancy were included in the analysis.

### Assessment of maternal iron status

We measured plasma ferritin, an established biomarker of body iron [14], to assess the iron status of mothers at an average gestational week (GW) of 14 (mean = 14, total range = 8–24; 5^th^-95^th^ percentiles = 12–17) and 30 (mean = 30, total range = 22–40; 5^th^-95^th^ percentiles = 28–34). Participating women were prescheduled to visit health centers, and their venous blood samples were collected. The blood samples were centrifuged, followed by separation of plasma and then stored at −70°C in freezers in Matlab. Then, they were shipped on dry ice to the University of California, Davis, where radioimmunoassays (Diagnostic Products, San Diego, CA, USA) were used to analyze the samples for plasma ferritin [15].

### Outcome variables

The outcome variables were neonatal birth weight and length. Approximately 40% had anthropometric assessment at the health clinics where delivery took place [16]. In cases of home delivery, a birth notification system was established for trained health workers to retrieve anthropometric assessments, typically within 72 hours of birth. For measurements taken more than 72 hours after delivery (13%), the birth weight and length were adjusted as described elsewhere [17]. Birth weights were measured with electronic scales (UNICEF Uniscale; SECA, Hamburg, Germany; precision of 10 g) that were calibrated daily. Birth length was measured with a locally made, wooden infantometer with 1 mm precision. The health workers periodically received refresher training on anthropometric measurements, and an independent team repeated the anthropometric measurements for a random sample of approximately 5% of the newborns [16].

### Covariates

Information on maternal age, weight, height, parity, education, socioeconomic status (SES), and date of delivery and on newborn sex were obtained from the MINIMat databases. The maternal body mass index (BMI, kg/m^2^) was calculated based on the woman’s weight and height recorded at the time of enrollment (at ~9 weeks gestation). Pregnancy weight gain (in kg) was calculated by subtracting the weight measured at enrollment from the weight measured at GW30. The gestational age (weeks) was calculated by subtracting the date of the last menstrual period (LMP) from the date of the pregnancy outcome. The LMP dates were obtained by interviewing the women during the process of pregnancy identification. Gestational age was also assessed by ultrasonography. Parity was defined as the number of live- or stillborn children before the current pregnancy. Maternal education was categorized according to the number of completed years of formal schooling: no education, 1–5 years, and ≥6 years. SES was based on wealth scores, derived mainly from ownership of a number of durable household assets by using principal component analysis [18], and categorized into tertiles (poorest, middle class, and wealthiest). The hemoglobin concentrations (Hb; g/L) were measured in venous blood by a HemoCue photometer (HemoCue AB, Angelholm, Sweden) at GW14 and GW30. Anemia was defined if Hb <110 g/l (8) and iron deficiency was defined with ferritin <12 μg/l [19]. We also determined whether food and micronutrient supplementation for food (two groups), micronutrient supplementation (three groups), or the combination of both (six groups) affected our studied association.

### Statistical analyses

We assessed the bivariate associations using Spearman’s rank correlation coefficient (r_s_), Kruskal–Wallis test, or *χ*^2^ tests, depending on the type of data. The associations of serum ferritin in tertiles with size at birth were assessed by multivariable-adjusted linear regression. The models were adjusted for variables that were significantly associated with exposure and outcome (*p *< 0.05), that changed the effect size estimates by 10% or more, or that are known or proposed to affect size at birth. We tested for collinearity between covariates, and those that were strongly associated (r_s_>0.60) were not included in the same model. There were strong correlations between maternal age and parity, as well as between SES and maternal education. Therefore, only parity and education were included in the models, as they had the largest influence on the estimates. Furthermore, we estimated the odds ratios (ORs) and 95% confidence intervals (CIs) of low birth weight (LBW) (<2,500 g) and preterm birth (gestational age <37 weeks) in relation to tertiles of maternal plasma ferritin at GW14 and GW30.

Statistical significance was considered when a 95% CI did not include zero or at a *p*-value <0.05 (two-sided). The statistical analyses were conducted using SPSS (version 24.0, IBM Corporation, USA) and STATA (version 14; STATA Corp, College Station, TX, USA).

## Results

**Figure 1.**
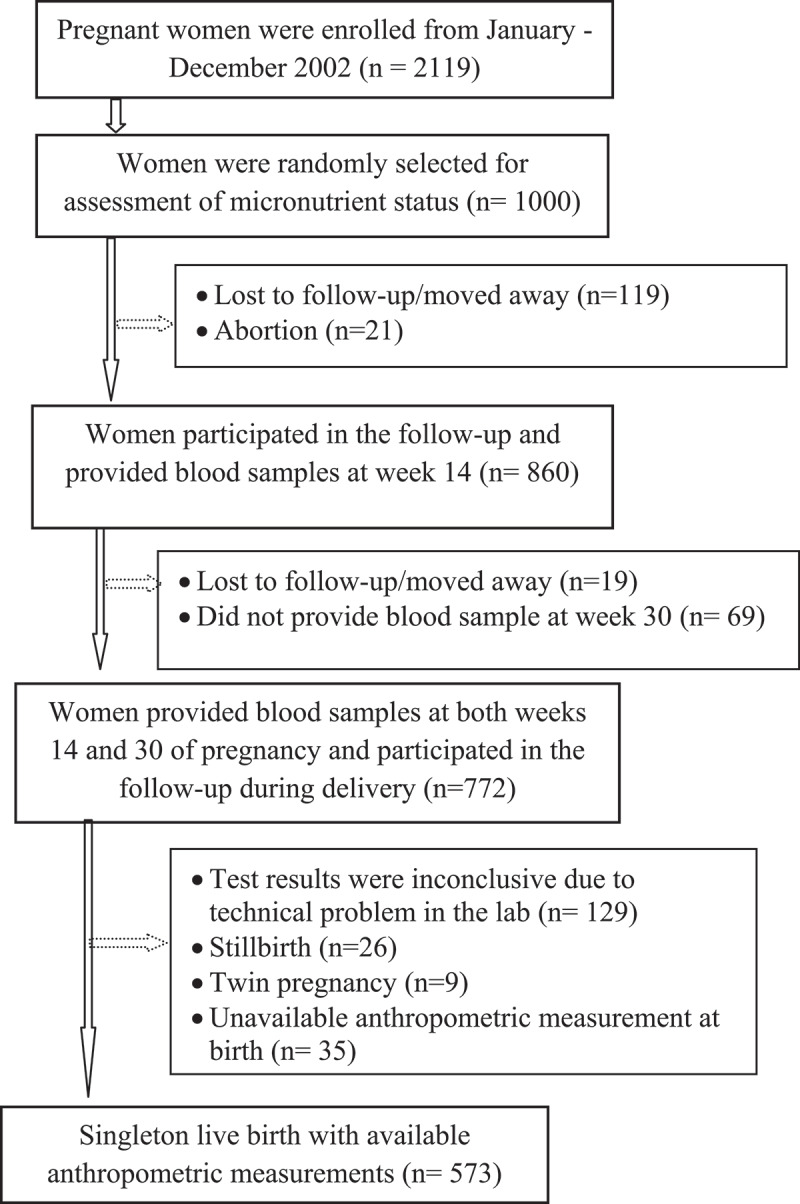
Flowchart of participating women

Our analysis involved pregnant women enrolled in the MINIMat trial during one calendar year from January to December 2002 (n = 2119; Figure 1). From this cohort, 1000 women were randomly selected for assessment of micronutrient status during pregnancy. Among them, the 129 first selected samples, analyzed in a separate first laboratory run, were discarded as the assay kits used were found to provide unreliable results, 69 women did not provide blood samples at 30 weeks of pregnancy, and 229 women were excluded due to loss of follow-up and adverse fetal outcome or anthropometric data was not available. The final analytic sample included 573 women with valid blood analyses available at both 14 and 30 weeks.

The sociodemographic characteristics of the participating mother-newborn dyads are presented in Table 1. The mean age of the mothers was 26 years (range 14–44 years). Approximately 33.1% were nulliparous, and 47% had 1–2 children. Almost one-third of the women had BMI<18.5 kg/m^2^ at 8 weeks. The mean gestational age at birth was 39 weeks (range 30–43 weeks), and 53% of the newborns were boys. The mean birth weight was 2,670 g (range 1,532–4,150 g), approximately 33% of the newborns weighed <2500 g (low birth weight, LBW), and 9.4% were preterm (<37 weeks). There were no significant differences in background characteristics (maternal age, BMI, education, parity, or SES) between those included (n = 573) and those excluded (n = 427). Additionally, we compared the background characteristics of the women included in the analysis (n = 573) and the women excluded due to discarded samples (n = 129). We did not find any significant difference between the two groups.

**Table 1.** Background characteristics of the mothers and their children (*n* = 573)

Characteristics	Number	Mean± SD or percentage	Median	5th–95th percentile
***Mothers***				
Age (years)	573	26 ± 6.1	26	18.0–37.0
Height (cm) at GW8 (kg)	573	150 ± 5.5	150	142 − 160
Weight at GW8 (kg)	573	51 ± 70	44.2	34.9–59.5
BMI at GW8 (kg/m^2^)	573	20.0 ± 2.7	19.6	16.2–25.4
Pregnancy weight gain (kg)^a^	569	5.4 ± 2.5	5.3	1.6–9.5
Education (years)				
No education	182	31.8%		
1–5	124	21.6%		
6 or more	267	46.6%		
Socioeconomic status (SES)				
Poorest	179	31.2%		
Middle class	198	34.6%		
Wealthiest	196	34.2%		
Parity				
Nulliparous	182	31.8%	1.5 ± 1.4	0.433
1–2	275	48.0%		
3 or more	116	20.2%		
P-ferritin (µg/L) at GW14	573	42.5 ± 29.1	35.6	11.1–97.6
P-ferritin (µg/L) at GW30	573	20.0 ± 14.4	16.7	4.6–49.4
Hb (g/L) at GW14	573	117 ± 12.2	116	100–139
Hb (g/L) at GW30	573	115 ± 11.8	115	97–135
***Newborns***				
Sex				
Boy	305	53.2%		
Girl	268	46.8%		
Gestational age at birth (weeks)	573	38.7 ± 2.2	39	35–42
Weight (g)	573	2,682 ± 400	2,670	2042–3400
Length (cm)	573	47.9 ± 2.2	48	44–51.8

SD: standard deviation; GW: gestational week; Hb: hemoglobin.

^a^Weight gain between GW8 and GW30.

The mean plasma ferritin concentration was 43 µg/L (range 2.4–201 µg/L) at GW14 and 20 µg/L (range 1.0–96 µg/L) at GW30. Of the 573 women, 29% were anemic (Hb <110 g/L; n = 166), and only 7% (n = 41) had iron deficiency (plasma ferritin <12 µg/L) at 14 weeks. Although the proportions of anemia were similar between GW14 and GW30 (34%), iron deficiency was higher (32%; n = 183) at GW30. However, only 68 women (12%) had both Hb <110 g/l and plasma ferritin <12 µg/L, and 125 (22%) women had anemia at GW30. The proportions of LBW babies were significantly higher among women in the highest ferritin tertile at GW30 (42%, 35%, and 23% in the highest, intermediate, and lowest tertiles, respectively; p = 0.020).

### Association between plasma ferritin at GW14 or GW30 and infant size at birth

In the multivariable-adjusted linear regression analyses (Table 2), birth weight was lower (B = −70; 95% CI: −147, 7.6; *p *= 0.077) among the newborns of women in the highest tertile of plasma ferritin at GW14 (median = 64 µg/L) compared to women in the lowest tertile (median = 17 µg/L). There was no association between plasma ferritin at GW14 and birth length (Table 2).

**Table 2.** Multivariable linear regression analysis of infants**’** size at birth (birth weight and length) in relation to plasma ferritin concentration (µg/L) categorized in tertiles at 14 weeks and 30 weeks of pregnancy (*n* = 573)

	Birth weight		Birth length	
	Β coefficient	95% CI	Β coefficient	95% CI
**Plasma ferritin at GW14**				
***Crude model***				
<25	Reference		Reference	
25–45	23	−58, 105	0.02	−0.43, 0.47
>45	− 43	−124, 58	−0.18	−0.62, 0.26
***Adjusted model* ^a^**				
<25	Reference		Reference	
25–45	3.8	−75, 83	0.09	−0.53, 0.35
>45	− 70	−146, 7.6	−0.31	−0.74, 0.126
**Plasma ferritin at GW30**				
***Crude model***				
<12	Reference		Reference	
12–21	−18	−99, 63	0.15	−0.29, 0.60
>21	− 74	−156, 8.54	−0.21	−0.66, 0.23
***Adjusted model* ^a^**				
<12	Reference		Reference	
12–21	−31	−109, 48	0.07	−0.37, 0.51
>21	−93	−172, −14*	−0.33	−0.77, 0.11

^a^Adjusted for maternal BMI, education, parity, and newborn sex.

*Statistically significant.

The adjusted model revealed a significant association (Table 2) between maternal plasma ferritin at GW30 and birth weight. The newborns of women in the highest tertile of plasma ferritin (median = 29 µg/L) had on average a 93-gm lower birth weight (95% CI: −172, –14; *p *= 0.021) than the newborns of women in the lowest tertile of plasma ferritin level (median = 8 µg/L). When plasma ferritin concentration was analyzed as a continuous variable (natural log-transformed to obtain normally distributed residuals with a homogeneous variance), in the multivariable-adjusted model, plasma ferritin at GW30 was inversely associated with birth weight (B = −57, 95% CI −102, −12; *p *= 0.014). There was no association between plasma ferritin at GW30 and birth length (Table 2).

In the multivariable logistic regression model (Table 3), the odds of LBW were more than two times higher (OR = 2.27; 95% CI: 1.40, 3.67) among women in the highest ferritin tertile at GW30 than among women in the lowest tertile. In this cohort, supplementation was given from 14 weeks until delivery. Consequently, the supplementation provided to those women who did not deliver preterm might have affected birthweights. However, the odds of LBW in term babies (GW 37 or more) were similar (OR = 2.63; 95% CI: 1.51, 4.57) among women in the highest ferritin tertile at GW30 than among women in the lowest tertile.

**Table 3.** Multivariable logistic regression analysis of the association between plasma ferritin concentration (µg/L) categorized in tertiles at 14 weeks and 30 weeks of pregnancy (*n* = 573) and low birth weight (LBW) infant and preterm birth

	Low birth weight		Preterm birth	
	OR	95% CI	OR	95% CI
**Plasma ferritin at GW14**				
***Crude model***				
<25	1.00		1.00	
25–45	0.96	0.62, 1.50	1.32	0.65, 2.72
>45	1.27	0.82, 1.94	1.24	0.61, 2.54
***Adjusted model*^a^**				
<25	1.00		1.00	
25–45	1.10	0.68, 1.76	1.33	0.63, 2.79
>45	1.50	0.95, 2.36	1.33	0.64, 2.77
**Plasma ferritin at GW30**				
***Crude model***				
<12	1.00		1.00	
12–21	1.34	0.85, 2.11	1.00	0.47, 2.15
>21	1.87	1.20, 2.93*	1.71	0.85, 3.46
***Adjusted model*^a^**				
<12	1.00		1.00	
12–21	1.49	0.92, 2.42	0.87	0.40, 1.92
>21	2.27	1.40, 3.67*	1.65	0.80, 3.38

^a^Adjusted for maternal BMI, education, parity, and newborn sex.

*Statistically significant.

There was no significant association between plasma ferritin at GW14 and LBW. We could not find any association between plasma ferritin at GW14 or GW30 and preterm birth. We also evaluated the association between Hb concentration during pregnancy and low birth weight infants. We did not observe any association of Hb concentration at GW14 (OR = 1.01; 95% CI: 0.98, 1.02) or GW30 (OR = 1.00; 95% CI: 0.98, 1.01) with LBW.

## Discussion

In this prospective cohort study, we found a negative association between maternal plasma ferritin at GW30 and birth weight. The women in the highest tertile of plasma ferritin had babies weighing on average 93 g less than those born to women in the lowest tertile. This reduction has significant public health implications, as reflected by the two times higher odds of LBW at the same level of ferritin exposure. We did not find a similar, significant association between plasma ferritin at GW14 and birth weight.

Our findings are consistent with previous studies reporting the associations between high maternal ferritin, measured between GW26 and GW36, and preterm birth [20,21], fetal growth restriction [10], or birth weight [22,23]. On the other hand, Vazirinejad and colleagues reported a positive correlation between maternal ferritin and both birth weight and length [24]. Of note, in Vazirinejad’s study, blood samples for ferritin were collected just before delivery, a period at which fetal growth would have been completed.

The inverse association between plasma ferritin concentration in the highest tertile (median = 29 µg/L) and birth weight was evident in late pregnancy, and the explanation may be multifold. Placental oxidative stress due to a systemic inflammatory response during the third trimester and activation of maternal endothelial cells all produce high amounts of circulating reactive oxygen species (ROS) which may cause intrauterine growth retardation of the fetus [25,26]. Iron is present at high levels in the placenta and is a necessary cofactor for the formation of free radicals. In contrast, iron deficiency results in defective mitochondrial function and mitochondrial DNA damage, which increase ROS formation [27]. Studies have suggested that iron excess causes free radical damage due to oxidative stress during pregnancy that can lead to functional disturbances [28]. It has been postulated that maternal iron build-up could increase blood viscosity, resulting in impaired uteroplacental circulation and, eventually, impaired fetal growth [29]. This putative mechanism could be more pronounced among iron-replete women taking supplementation [30]. Interestingly, recent studies reported that routine iron supplementation in nonanemic mothers is not beneficial for pregnancy outcome, and they suggested using iron supplementation cautiously [20,31]. The high prevalence of hemoglobin E (HbE) and β- thalassemia in Bangladesh [6] could have contributed to the appreciable proportion of non-iron deficiency anemia and iron excess in the women at GW30. However, thalassemia screening was not undertaken among the studied women.

Several studies indicated that a high ferritin concentration in the third trimester correlates with preterm delivery, probably due to subclinical maternal infection [20,22,32]. Moreover, plasma ferritin is a positive acute phase reactant that may increase in association with cardiometabolic conditions such as hypertension, gestational diabetes mellitus (GDM), and preeclampsia. As part of MINIMat trial, blood pressure, urinary albumin and glucose were monitored. However, the purpose was not to strictly diagnose gestational diabetes or pre-eclampsia, rather it was to detect danger sign(s) in any randomized mother, so that she/they could have been referred for appropriate care. Consequently, we did not use those data in the analysis.

Nevertheless, elevated plasma ferritin in our participants was not influenced by acute infection or inflammation, which was confirmed by C-reactive protein (CRP) levels in early and late pregnancy. Only 4% of the MINIMat women had elevated CRP levels (10 mg/L) at GW14 and 7% at GW30 [33].CRP was analyzed semi-quantitatively by radial immunodiffusion (The Binding Site, San Diego, CA, USA) in women with a cutoff of 10 mg/L [33].

It is noteworthy that for mothers in the highest ferritin tertile, the median plasma ferritin at GW30 was less than half of that at GW14 (29 µg/L versus 64 µg/L). This finding was in line with Goldenberg et al. who reported a decline in the mean plasma ferritin level from 41 pg/L at 19 weeks to 18 pg/L at 36 weeks [22]. We believe that physiological hemodilution underlies such a reduction. Whereas it may appear counterintuitive that the association with birth weight was significant for plasma ferritin at GW30 but not at GW14, the fall in plasma ferritin among participants of this study could be of a lesser magnitude than that usually occurring in the third trimester because of the iron supplementation they received as part of the trial.

The main strengths of this study include its prospective, population-based design with a cohort of pregnant women who did not smoke or consume alcohol. We considered multiple potential confounders, including maternal age, parity, BMI, education, SES, and child sex. All data were collected by trained health workers and technicians. We used plasma ferritin as an indicator of iron status, which is more specific than Hb, albeit sensitive to concurrent infection. One limitation was the lack of data on plasma ferritin for a considerable proportion of participants (n = 427), which would have strengthened our evaluation of the association between ferritin and size at birth. To address the natural dip in birth weight after the first day [34], we adjusted birth weights for babies who were weighed more than 72 hours after birth.

## Conclusion

In the current study, we observed an inverse association between higher maternal ferritin in late pregnancy and infant birth weight. The magnitude of the reduction in birth weight (B = −93 gm; 95% CI: −172, –14) corresponds to approximately 25% of the standard deviation of the mean birth weight and reflects a similar effect of tobacco smoking [35] or intervention by food supplementation [36]. Therefore, the study results have significant public health implications. The observed birth weight reduction has future short- and long-term health impacts on children and warrants further studies to explore the causal link of the association between plasma ferritin level and birth weight in other settings.

## Data Availability

The datasets during and/or analyzed during the current study available from the corresponding author on reasonable request.

## Paper context

Ferritin level is utilized to assess body iron status. Studies raise concern regarding the association of high maternal plasma ferritin with impaired early-life growth and development. We explored the association between plasma ferritin levels during pregnancy and infants’ weight and length at birth in a population-based cohort in rural Bangladesh. An inverse association between high plasma ferritin in the last trimester and birth weight was observed. This may underlie short- and long-term health consequences among the children.
